# *Chlamydia trachomatis* Plasmid Protein pORF5 Up-Regulates ZFAS1 to Promote Host Cell Survival via MAPK/p38 Pathway

**DOI:** 10.3389/fmicb.2020.593295

**Published:** 2020-12-17

**Authors:** Yating Wen, Hongliang Chen, Fangzhen Luo, Lanhua Zhao, Mingyi Shu, Shengmei Su, Yuqi Zhao, Qiulin Huang, Zhongyu Li

**Affiliations:** ^1^Institute of Pathogenic Biology, Hengyang Medical College, Hunan Provincial Key Laboratory for Special Pathogens Prevention and Control, Hunan Province Cooperative Innovation Center for Molecular Target New Drug Study, University of South China, Hengyang, China; ^2^Department of General Surgery, First Affiliated Hospital of University of South China, Hengyang, China

**Keywords:** *Chlamydia trachomatis*, pORF5, long non-coding RNA, ZFAS1, MAPK

## Abstract

Long non-coding RNAs (lncRNAs) have been demonstrated to play essential roles in many diseases. However, few studies have shown that lncRNAs take part in the pathogenesis of *Chlamydia trachomatis* (*C. trachomatis*). Here, we used a lncRNA microarray to detect the global lncRNA expression profiles in HeLa cells transfected with pORF5 plasmid protein, an important virulence factor for *C. trachomatis*. The differentially expressed lncRNAs and mRNAs screened by microarray were selected for validation by quantitative real-time PCR. The up-regulated lncRNA zinc finger antisense 1 (ZFAS1) was presumed to involved in MAPK pathways by bioinformatics analysis. Inhibition of ZFAS1 decreased the apoptotic rate of pORF5 and reduced the infectivity of *C. trachomatis*, and MAPK/p38 pathway was involved in anti-apoptotic effect induced by ZFAS1. Therefore, the present study confirmed that pORF5 up-regulates ZFAS1 to promote host cell survival via MAPK/p38 pathway and influences the infectivity of *C. trachomatis.*

## Introduction

*Chlamydia trachomatis* (*C. trachomatis*) is an obligate intracellular, gram-negative pathogen responsible for many diseases such as trachoma and sexually transmitted diseases. Genital infection of *C. trachomatis* is most common, however, it usually leads to pelvic inflammatory disease, tubal factor infertility or ectopic pregnancy because of the scarring of the reproductive tract induced by asymptomatic infection (Zhong, 2018). This pathogen alternates between two morphological forms, the infectious elementary body (EB) and the intracellular, non-infectious reticulate body (RB) (Bastidas et al., 2013). EBs enter host cells and differentiate into RBs, then replicate in a special niche termed inclusion and utilize numerous strategies to survive in the host cells (Chen et al., 2019).

To interact with host, *C. trachomatis* delivers virulence proteins called effectors into the host cell by secretion system. These effector proteins affect host physiology to acquire nutrients, manipulate apoptotic pathways, and interfere with immune responses. pORF5 is the only secreted protein in eight plasmid-encoded proteins in *C. trachomatis* (Li et al., 2008). pORF5 has been showed to be an essential virulence factor for the pathogen, and strongly induce the production of inflammatory factors (Cao et al., 2015; Hou et al., 2019). Our previous studies also verified that pORF5 influenced the expression of host proteins (Zou et al., 2018). These alternated proteins take part in cellular processes including apoptosis and autophagy (Lei et al., 2017), indicating that pORF5 plays a key role in the pathogenesis of *C. trachomatis*.

Long non-coding RNA (lncRNA) is a kind of non-coding RNAs (ncRNAs) that the length over 200 nucleotides. Most studies have proved that they have essential roles in epigenetic regulation and a series of biological processes, including transcriptional regulation, intranuclear transport, post-transcriptional modifications, translation, splicing, differentiation, cell cycle control, and so on (Yao et al., 2019). Meanwhile, growing evidence implies that altered expression of lncRNAs could be closely related to genesis and progression of numerous diseases, especially in cancers and viral infections (Liu and Ding, 2017; Sun et al., 2020). Recently, numerous studies have provided evidence for the involvement of lncRNAs in bacterial infections, including *Mycobacterium tuberculosis*, *Helicobacter pylori*, *Listeria monocytogenes*, and so forth (Liu et al., 2018; Li M. et al., 2019; Xu et al., 2019). However, few studies have evaluated the alternation of lncRNAs in obligate intracellular bacterium *C. trachomatis*.

The molecular mechanisms underlying pathogenesis of *C. trachomatis* are still elusive, and lncRNAs may provide new insights into the potential mechanisms. Thus, we performed a microarray analysis to detect the global lncRNAs and mRNAs expression in pORF5-transfected HeLa cells, and tried to identify pORF5-related lncRNAs. Additionally, we confirmed that pORF5 could activate the p38 pathway by up-regulating ZFAS1, promoting the survival of host cells and the proliferation of *C. trachomatis*.

## Materials and Methods

### Cell Lines and Cell Culture

HeLa cells and HEK293T cells were cultured and maintained in DMEM (Dulbecco’s Modified Eagle Medium; Gibco, Karlsruhe, Germany) supplemented with 10% (v/v) fetal bovine serum (FBS; Gibco) at 37°C in 5% atmosphere. *C. trachomatis* used in this study was cultured as previous research (Li et al., 2008).

### Lentivirus Vector Construction and Transfection

The pORF5 gene was cloned into pHBLV-CMV-MCS-3FLAG-EF1-ZsGreen-T2A-PURO vector using HB-infusion^TM^ (HanBio Biotechnology Co., Ltd., Shanghai, China) according to the instructions of manufacturer. When cells fused about 70–80% in 100 mm plates, the cells were co-transfected with pHBLV-CMV-MCS-3FLAG-EF1-ZsGreen-T2A-PURO-pORF5 plasmid (or the pHBLV-CMV-MCS-3FLAG-EF1-ZsGreen-T2A-PURO control plasmid), the lentiviral packaging plasmid pSPAX2 and lentiviral envelope plasmid pMD2G. The fresh medium was added to incubate 2 days follow 6-h incubation. The medium was harvested at 72 h post-transfection and centrifugation for harvest the lentivirus vector. Stable clones were selected in DMEM medium containing puromycin (10 μg/mL) for 5 days.

### RNA Extraction and lncRNA Microarray

The total RNA was isolated from freshly harvested pORF5-transfected cells and GFP-transfected cells using TRIzol reagent (Invitrogen). We employed a human lncRNA microarray V4.0 (8 × 60 K, Arraystar, Rockville, MD, United States) containing approximately 40,173 lncRNAs and 20,730 coding transcripts to screen the differentially expressed lncRNAs and mRNAs. The protocol was listed as follows: First, total RNAs were collected from each time points and extracted by TRIzol; Second, purity and integrity of extracted total RNA were measured using a NanoDrop nd-1000 spectrophotometer; Third, complementary DNAs were labeled with an Arraystar RNA Flash Labeling Kit, purified with an RNeasy Mini Kit (Qiagen), and hybridized with lncRNA microarrays; Forth, microarrays were scanned by Agilent DNA microarray scanner (Agilent p/n G2565BA). Quantile normalization, data processing, and hierarchical clustering were performed with the GeneSpring GX v11.5.1 software package (Agilent Technologies). Differentially expressed lncRNAs and coding transcripts with statistical significance between the two groups were identified through *P*-value and fold change filtering.

### Quantitative Real-Time PCR

Total RNA extracted from cells by TRIzol reagent was subsequently reverse transcribed into cDNA by reverse transcription kit (Tiangen Biotech Co., Ltd., Beijing, China) according to the instruction of the manufacturer. The quantitative real-time polymerase chain reaction (qRT-PCR) was performed using the SYBR Green premix (Tiangen) in a LightCycler 96 apparatus (Roche, Basel, Switzerland). *18S rRNA* acts as an internal control. Reactions were performed in duplicate for each sample. Data were normalized as the ratio of lncRNA transcript to *18S rRNA* transcript. The relative expression level was calculated by the delta-delta-Ct method. Primers designed for validation were synthesized by Sangon (Sangon Biotech, Shanghai, China) and shown in Table 1.

**TABLE 1:** Primers designed for qRT-PCR validation of candidate lncRNAs.

**Gene Symbol**	**Forward**	**Reverse**
MAPKAPK3	GGACAGAAGTGTGCCCTGAAGC	GCCAGAAGCCTGCCAGTGATG
DDIT3	TCTGGCTTGGCTGACTGAGGAG	TCTGACTGGAATCTGGAGAGTGAGG
SESN2	TGCCACTCGCTCTCCTCCTTC	GTTCAACGGGTCCCTGCTTGG
FZD10	GCGAGGCAGCCATCCAGTTG	CGTACAGCGAGCACAGGAAGAAG
MAP3K13	AACTTGAAGACCGCTTGGCAGAG	TGTCAGAGAGCCCATCCGAGTG
NKRF	ACTCGTTGTATTCAGGCGTGTAAGAC	GGGATGTCGGCAGGAGGAATTTC
DKK-1	TACCAGACCATTGACAACTACC	TCCATTTTTGCAGTAATTCCCG
CASP2	ACAATAAAGATGGTCCTGTCTGCCTTC	ACATTGCTCAACACCAGTGCTAGG
H19	CGTGACAAGCAGGACATGACA	CCATAGTGTGCCGACTCCG
GAS5	GAGCAAGCCTAACTCAAGCCA	GACTCCACCATTTCAACTTCCA
RP11-42O15.3	GTTCTTCCTCATAGGAAAAATGGG	GGTTTTTACTAAGCCAAAGGGATAG
ZFAS1	CTGGCGATGGAATATGAGAGG	CTTTATGCAGGTAGGCAGTTAGAAA
BASP1-AS1	TGGAACCGTTGGATAAGAAGTG	GTCATTCATCACCTCGAGGCA
FGD5-AS1	GAGATCATTCAGAGAGACAGGTCG	AAGGACAAGGCACATCCTACG
CRHR1-IT1	TCTACCCTTGATGCTAACAATGTTC	TACAGTCCAGTTGCAGAAACTTTGT
CALML3-AS1	TGGCGAAAGGATTGTTGGAA	ACAACCCAGGCAAATCAAACAG
*18S rRNA*	CGCTCGCTCCTCTCCTACTT	CGGGTTGGTTTTGATCTGATAA

### Bioinformatics Analysis

To obtain the functions of differentially expressed coding transcripts, Gene ontology (GO) analysis was identified by DAVID Bioinformatics Resources^1^, and the significant GO terms were identified as a *P*-value < 0.05. Pathways analysis was used by Kyoto Encyclopedia of Genes and Genomes (KEGG) database^2^. Protein-protein interaction net (PPI) network was performed by STRING^3^ and visualized by Cytoscape 3.7.1^4^. Further analysis of KEGG pathways was used by Metascape^5^ (Zhou et al., 2019).

### RNA Interference

Small-interfering RNA (siRNA) against ZFAS1 and the negative control siRNA oligos were synthesized by RiboBio (RiboBio Co., Ltd., Guangzhou, China). The siRNA oligos were transfected into HeLa cells at 50 nM by Lipofectamine 2000 (Invitrogen). The sequence of siRNA oligo used in this study is 5′-GAGGGGAGCGGACCGCGGG-3′.

### Apoptosis Assay

Hoechst staining and flow cytometry analysis were applied to measured apoptosis. Cells were incubated with Hoechst 33258 (Beyotime Biotech, Nanjing, China) for 1 h after fixed with 4% paraformaldehyde. The apoptotic cells were observed by fluorescence microscope (Nikon, Japan). Apoptotic cells were also detected with Annexin V-APC/PI apoptosis detection kit (MULTI SCIENCES, Hangzhou, China). Briefly, cells were trypsinized and harvested after appropriate treatment. Harvested cells were then incubated with Annexin V-APC and propidium iodide (PI) for 15 min in the dark according to the manufacturer’s instructions. The stained cells were determined by flow cytometer (FACSCalibur, BD, United States).

### Western Blot Analysis

Total proteins of the cells were isolated with RIPA buffer containing protease and phosphatase inhibitors on ice for 20 min. After centrifugation, the supernatants were boiled at 100°C for 5 min and were resolved by 12% SDS-PAGE. Then the proteins transferred to a PVDF membrane (0.22 μm; Millipore, Billerica, MA, United States). The membranes were blocked with 5% non-fat milk in TBST buffer (25 mM Tris–HCl, 125 mM NaCl, 0.1% Tween 20) for 2 h at room temperature, and incubated with primary antibodies overnight. pORF5 and specific primary anti-mouse antibody was purified and stored as described according to published work (Li et al., 2008). The anti-rabbit antibodies to β-actin, ERK1/2, p-ERK1/2, JNK, p-JNK, p38, p-p38, cleaved caspase 3 and caspase 3 were acquired from Cell Signaling Technology (Danvers, MA, United States). The inhibitors to ERK (PD98059), JNK (SP600125), and p38 (SB202190) were purchased from Sigma (St. Louis, MO, United States). Finally, the membrane was incubated with horseradish peroxidase (HRP)-conjugated goat anti-rabbit or anti-mouse IgG (Abcam, Cambridge, United Kingdom) for 1 h at 37°C. The results were visualized using an enhanced chemiluminescence Western blot system G:Box Chemi XXX9 (Syngene, Cambridge, United Kingdom). The densities of protein bands were analyzed by Quantity One (Bio-Rad, United States).

### Statistical Analysis

All data were presented as mean ± SD. Data were analyzed and visualized with SPSS14.0 and GraphPad Prism 5.0. The statistical significance of differences between different groups was analyzed with two-tailed Student’s test. Statistical significance was set at *P* < 0.05.

## Results

### Over-Expression of pORF5 in Transfected HeLa Cells

**FIGURE 1:**
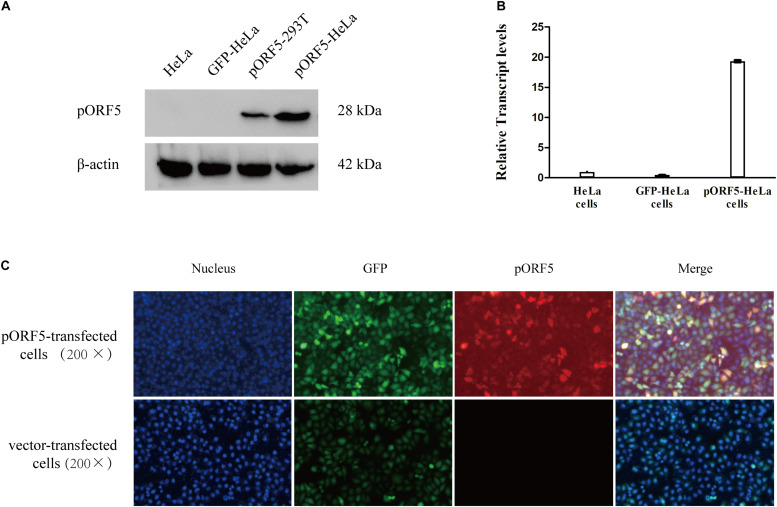
Over-expression of pORF5 in transfected HeLa cells. **(A,B)** The protein and mRNA expression levels of pORF5 were tested by western blot and qRT-PCR, respectively. **(C)** The expression of pORF5 detected by IFA. DNA (blue) was stained with Hoechst 33258, GFP (green) was expressed by transfected plasmid. pORF5 (red) was stained with Cy3 (200×).

To explore the role of pORF5 in the pathogenesis of *C. trachomatis*, pORF5 gene was inserted into lentivirus and expressed in the HeLa cells. As shown in Figure 1, compared with the vector-HeLa cells, both mRNA and protein expression levels of pORF5 were remarkably up-regulated in the pORF5-transfected HeLa cells. The pORF5-transfected HeLa cells and vector-cells were used for further experiments.

### Differential Expression of lncRNAs and mRNAs in pORF5-Transfected HeLa Cells

In order to obtain a whole landscape of differentially expressed lncRNAs and mRNAs induced by pORF5, we performed a microarray analysis between pORF5-transfected HeLa cells and vector-transfected HeLa cells using Arraystar Human LncRNA Array v4.0. After normalization of the raw data, the expression profiles of 40,173 lncRNAs and 20,730 mRNAs in pORF5-transfected cells and control cells were obtained. The normalized intensity values of lncRNAs and mRNAs between pORF5-transfected HeLa cells and control cells were almost the same. Supplementary Figures 1A,B showed the heat maps of lncRNAs and mRNAs.

In total, we identified 271 dysregulated lncRNAs (fold change ≥ 2), of which 153 (56.5%) were upregulated and 118 (43.5%) were downregulated in pORF5-transfected HeLa cells compared to control cells. Eleven lncRNAs exhibited a high fold change (over four-fold) (6 increased and 5 decreased) (Supplementary Figure 1C). 223 mRNAs were differentially expressed in pORF5-transfected cells compared to the control cells, among which 92 mRNAs were increased and 131 mRNAs were decreased (fold change ≥ 2) (Supplementary Figure 1D). There were 17 mRNAs exhibiting a high fold change (over four-fold) (10 increased and 7 decreased). The top 20 differentially expressed lncRNAs were summarized in Tables 2, 3.

**TABLE 2:** Top 20 dysregulated mRNAs detected using microarray.

**Upregulated mRNAs**	**Genes**	**Fold change (pORF5 vs. GFP)**	**Downregulated mRNAs**	**Genes**	**Fold change (pORF5 vs. GFP)**
NM_004635	MAPKAPK3	58.8758631	NM_006308	HSPB3	10.1625828
NM_004083	DDIT3	10.1309461	NM_000735	CGA	6.7978744
NM_019113	FGF21	10.1053039	NM_182559	TMPRSS12	5.5931836
NM_024111	CHAC1	7.8812611	NM_014714	IFT140	5.2781999
NM_001216	CA9	5.2224179	NM_005346	HSPA1B	4.832871
NM_020704	STRIP2	4.9354783	NM_006597	HSPA8	4.3904125
NM_007197	FZD10	4.8605767	NM_002155	HSPA6	4.3050497
NM_006573	TNFSF13B	4.2762266	NM_033049	MUC13	3.9187893
NM_003714	STC2	4.1094178	NM_012242	DKK1	3.4686116
NM_006472	TXNIP	4.0062349	NM_016356	DCDC2	3.4438765
NM_001902	CTH	3.7109228	NM_019001	XRN1	3.3692199
NM_030767	AKNA	3.4991294	NM_012347	FBXO9	3.2901287
NM_001291556	PCK2	3.4559444	NM_005527	HSPA1L	3.2773751
NM_032632	PAPOLA	3.3306713	NM_000900	MGP	3.174398
NM_002606	PDE9A	3.2654398	NM_021016	PSG3	3.1134631
NM_004345	CAMP	3.2006005	NM_002783	PSG7	2.9754882
NM_000728	CALCB	3.1401514	NM_020632	ATP6V0A4	2.9367592
NM_021158	TRIB3	3.1155829	NM_005559	LAMA1	2.9238575
NM_145699	APOBEC3A	3.0654269	NM_014872	ZBTB5	2.9216983
NM_001017930	DCAF8L1	3.0476778	NM_182536	GKN2	2.9207699

**TABLE 3:** Top 20 dysregulated mRNAs detected using microarray.

**Upregulated mRNAs**	**Genes**	**Fold change (pORF5 vs. GFP)**	**Downregulated mRNAs**	**Genes**	**Fold change (pORF5 vs. GFP)**
NM_004635	MAPKAPK3	58.8758631	NM_006308	HSPB3	10.1625828
NM_004083	DDIT3	10.1309461	NM_000735	CGA	6.7978744
NM_019113	FGF21	10.1053039	NM_182559	TMPRSS12	5.5931836
NM_024111	CHAC1	7.8812611	NM_014714	IFT140	5.2781999
NM_001216	CA9	5.2224179	NM_005346	HSPA1B	4.832871
NM_020704	STRIP2	4.9354783	NM_006597	HSPA8	4.3904125
NM_007197	FZD10	4.8605767	NM_002155	HSPA6	4.3050497
NM_006573	TNFSF13B	4.2762266	NM_033049	MUC13	3.9187893
NM_003714	STC2	4.1094178	NM_012242	DKK1	3.4686116
NM_006472	TXNIP	4.0062349	NM_016356	DCDC2	3.4438765
NM_001902	CTH	3.7109228	NM_019001	XRN1	3.3692199
NM_030767	AKNA	3.4991294	NM_012347	FBXO9	3.2901287
NM_001291556	PCK2	3.4559444	NM_005527	HSPA1L	3.2773751
NM_032632	PAPOLA	3.3306713	NM_000900	MGP	3.174398
NM_002606	PDE9A	3.2654398	NM_021016	PSG3	3.1134631
NM_004345	CAMP	3.2006005	NM_002783	PSG7	2.9754882
NM_000728	CALCB	3.1401514	NM_020632	ATP6V0A4	2.9367592
NM_021158	TRIB3	3.1155829	NM_005559	LAMA1	2.9238575
NM_145699	APOBEC3A	3.0654269	NM_014872	ZBTB5	2.9216983
NM_001017930	DCAF8L1	3.0476778	NM_182536	GKN2	2.9207699

The differentially expressed lncRNAs were widely distributed among all chromosomes except for sex chromosomes X and Y (Supplementary Figure 2A). These dysregulated lncRNAs were divided into five groups according to the association with well-annotated protein-coding genes, including natural antisense (13.28%), intronic antisense (5.90%), intron sense-overlapping (4.06%), exon sense-overlapping (2.21%), intergenic (69.01%), and bidirectional (5.54%) (Supplementary Figures 2B,C).

### Validation of the lncRNA and mRNA Expressions

To validate the reliability of the microarray results, eight differentially expressed lncRNAs (GAS5, RP11-42O15.3, H19, ZFAS1, BASP-AS1, FGD5-AS1, CRHR1-IT1, and CALML3-AS1) and six differentially expressed mRNAs (DDIT3, SESN2, FZD10, MAP3K13, NKRF, and DKK-1) predicated by microarray were selected for validation by RT-qPCR, and were normalized by internal control *18S rRNA* expression. All these chosen lncRNAs and mRNAs were similar to those in the results shown in microarray analysis; lncRNAs including BASP-AS1, FGD5-AS1, CRHR1-IT1 and CALML3-AS1, and mRNAs including MAP3K13, NKRF, and DKK-1 were down-regulated, whereas lncRNAs including GAS5, RP11-42O15.3, H19, and ZFAS1, and mRNAs including DDIT3, SESN2, and FZD10 were up-regulated in pORF5-transfected cells (Supplementary Figure 3). These results indicated that the expression alternations at the transcriptional level were consistent with microarray.

### Gene Ontology and Pathway Analysis for the Potential Functionalities of Differentially Expressed mRNAs

Gene ontology analysis for all the aberrant expressed mRNAs was used to identify the function of coding transcripts. Previous studies demonstrated that lncRNAs could regulate the adjacent or overlapping coding genes expressions (Spurlock et al., 2015). These coding genes might provide insight into these differentially expressed lncRNAs. To evaluate the enrichment of the differentially expressed mRNAs in GO terms and pathways, GO and KEGG analysis were conducted, which implied that the most significantly enriched biological processes of up-regulated genes in pORF5-transfected cells were observed with the following terms: response to the endoplasmic reticulum (ER) stress, response to unfolded protein, endoplasmic reticulum unfolded protein response (Supplementary Figure 4A), and cell components concentrated on microvillus membrane, extracellular region, extracellular matrix (Supplementary Figure 4C). The molecular functions mainly belonged to misfolded protein binding, unfolded protein binding, and protein binding involved in protein folding (Supplementary Figure 4E). The pathway analyses revealed that the most significantly enriched pathways of the up-regulated differentially expressed (DE) genes were alanine, aspartate and glutamate metabolism, biosynthesis of amino acids, and insulin resistance (Supplementary Figure 4G). The most significantly enriched biological processes of up-regulated DE genes in pORF5-transfected cells were protein refolding, chaperone cofactor-dependent protein refolding, and “*de novo*” post-translational protein folding (Supplementary Figure 4B), and cell components concentrated on the microtubule, supramolecular fiber, and supramolecular polymer (Supplementary Figure 4D). The molecular functions of down-regulated DE genes were associated with misfolded protein binding, unfolded protein binding, and protein binding involved in protein folding (Supplementary Figure 4F). The pathway analyses revealed that the most significantly enriched pathways of the down-regulated DE genes were antigen processing and presentation, legionellosis, and protein processing in ER (Supplementary Figure 4H). In addition, most of DE mRNAs are involved in the functional interaction according to a protein interaction network (PPI) (Supplementary Figure 5). These data provide compelling evidence for the underlying pathogenesis mechanisms of pORF5.

### Inhibition of ZFAS1 Reduces the Anti-apoptotic Effect of pORF5 and the Infectivity of *C. trachomatis*

**FIGURE 2:**
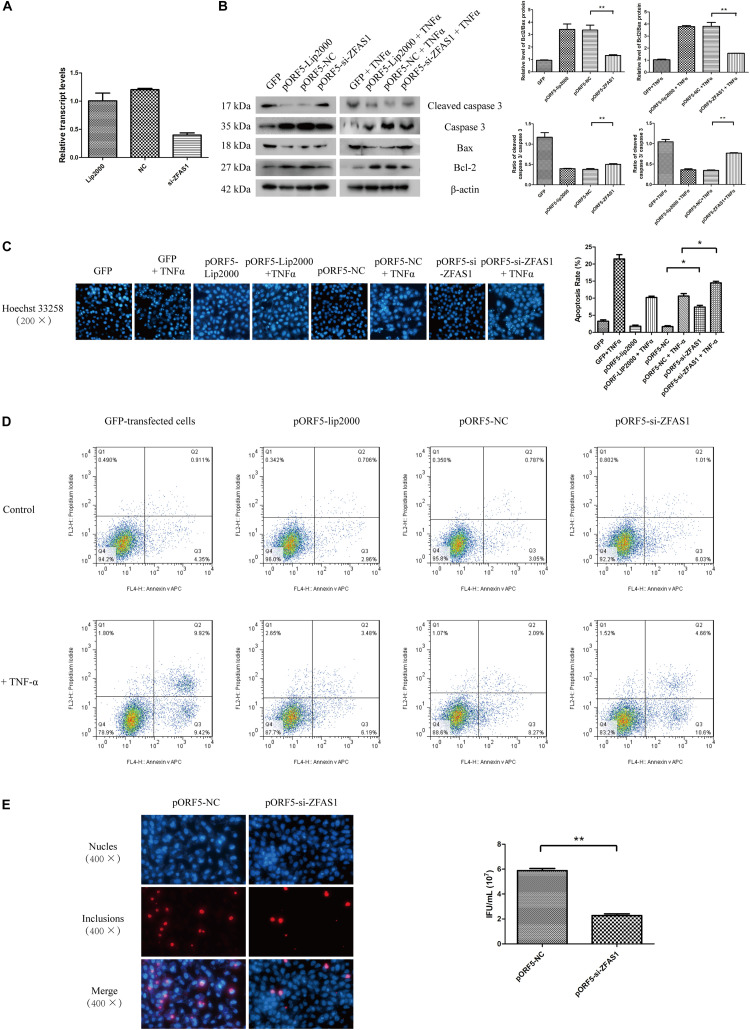
Inhibition of ZFAS1 reduces the anti-apoptotic effect of pORF5 and the infectivity of *C. trachomatis.* **(A)** Knockdown of lncRNA ZFAS1. **(B)** The detection of apoptosis in ZFAS1-interfered cells after the apoptotic induction by TNF-α. **(C)** The apoptotic rate (right panel) was detected by Hoechst staining (left panel). **(D)** The apoptotic rate was detected by FACS. **(E)** Infectivity of *C. trachomatis* was tested after the knockdown of ZFAS1. ^∗^*P* < 0.05; ^∗∗^*P* < 0.01.

ZFAS1 was previously observed to be tumor oncogene in hepatocellular carcinoma and esophageal squamous cell carcinoma (Guo et al., 2019; Li Z. et al., 2019). To preliminarily understand how ZFAS1 functions in pORF5-transfected cells, we down-regulated the expression of lncRNAs in pORF5-transfected cells. As shown in Figure 2A, the expression level of ZFAS1 was inhibited.

As pORF5 was identified to have a role in anti-apoptosis, we subsequently tested the expressions of classical molecules Bax, Bcl-2, caspase 3 and cleaved caspase 3 following the interference of ZFAS1. As shown in Figure 2B, compared to the negative control, the expression of Bax and cleaved caspase 3 in the ZFAS1-interfered group were increased while the expression of Bcl-2 and caspase 3 were reduced after the induction of apoptosis. And the apoptotic rate of cells in the ZFAS1-interfered group was remarkably increased following TNF-α treatment (Figure 2C). The anti-apoptotic effect of ZFAS1 was validated by FACS (Figure 2D). To explore the influence of ZFAS1 on *C. trachomatis*, ZFAS1-interfered cells were infected with *C. trachomatis* for 40 h and the progeny was harvested to infect HeLa cells. The infectivity of the ZFAS1-interfered group was dramatically reduced when compared to control group (Figure 2E). These observations indicated that pORF5-upregulated ZFAS1 is participated in antiapoptotic effect and influences the infectivity of *C. trachomatis*.

### MAPK/p38 Pathway Is Involved in Anti-apoptotic Effect of Up-Regulated ZFAS1

**FIGURE 3:**
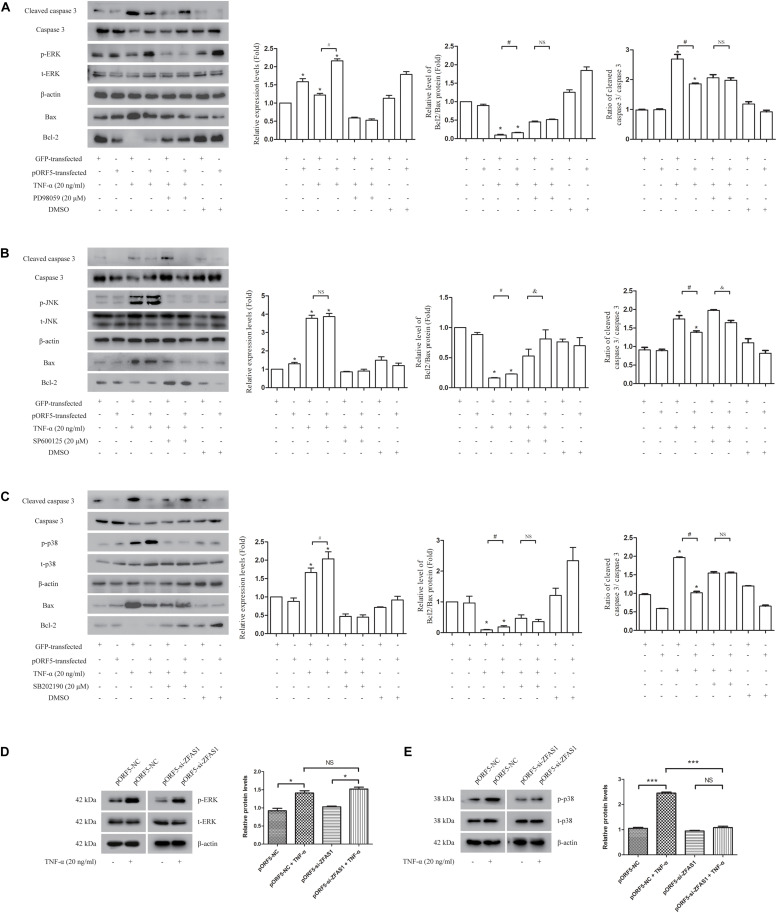
MAPK/p38 pathway is involved in anti-apoptotic effect of up-regulated ZFAS1. **(A–C)** The detection of ERK **(A)**, JNK **(B)**, and p38 **(C)** activation after the usages of TNF-α and corresponding inhibitors by western blot. Second panel is the gray scale of left molecules band (ERK, JNK, or p38). Third panel is the relative levels of Bcl2/Bax. Right panel is the ratio of cleaved caspase 3/caspase 3. **(D,E)** The detection of ERK **(D)** and p38 **(E)** activation after the inhibition of ZFAS1 by western blot. Right panel is the gray scale of left molecules band (ERK or p38). ^*,#,&^*P* < 0.05; ^∗∗∗^*P* < 0.001; NS, no significance.

The significantly differentially expressed mRNA moleculeMAPKAPK3 is one of the Serine/Threonine protein kinase, and acts as a mitogen-activated protein kinase-activated protein kinase. Thus, we next investigated the role of MAPK pathways including ERK, p38, and JNK in pORF5-transfected cells. After TNF-α induction in pORF5-transfected cells, the activation of three pathways was detected. As shown in Figure 3, the relative levels of ERK, p38, and JNK phosphorylation were (2.165 ± 0.052), (2.137 ± 0.140), and (3.864 ± 0.180) in pORF5-transfected cells, and the control cells were (1.222 ± 0.045), (1.600 ± 0.058), and (3.771 ± 0.171), respectively. The phosphorylation level of ERK and p38 in pORF5-transfected cells was higher than that of control cells (*P* < 0.05), but there was no significant change in the phosphorylation level of JNK in both cell lines (*P* > 0.05), suggesting that ERK and p38 pathways were involved in anti-apoptosis process of pORF5-transfected cells. Subsequently, cells were pretreated with ERK inhibitor (PD98059), JNK inhibitor (SP600125) and p38 inhibitor (SB202190), and the Bcl-2/Bax ratio and the cleaved caspase 3/caspase 3 ratio were detected after TNF-α induction. The results showed in Figure 3. The Bcl-2/Bax ratios were (0.454 ± 0.024) and (0.513 ± 0.019), and the cleaved caspase 3/caspase 3 ratios were (2.061 ± 0.189) and (1.978 ± 0.084) in the control cells and pORF5-transfected cells pretreated with ERK inhibitor. No significant difference could be observed in the Bcl-2/Bax ratio and the cleaved caspase 3/caspase 3 ratio between the two groups (*P* > 0.05). The Bcl-2/Bax ratio in control cells treated with JNK inhibitor (0.428 ± 0.057) was lower than that of pORF5-transfected cells (0.513 ± 0.019) (*P* < 0.05), but there was no significant difference between the two groups after treated with p38 inhibitor [(0.467 ± 0.110) vs. (0.380 ± 0.072), (*P* > 0.05)]. The cleaved caspase 3/caspase 3 ratio in control cells treated with JNK inhibitor (1.973 ± 0.022) was higher than that of pORF5-transfected cells (1.642 ± 0.061) (*P* < 0.05), while no significant difference between the two groups in p38 inhibitor-treated cells (*P* > 0.05). The above results indicated that inhibition of ERK and p38 pathways reduced the anti-apoptotic ability of pORF5-transfected cells, and pORF5 exerted anti-apoptotic effects through the MAPK/ERK and MAKP/p38 pathways. To further investigate the relationship between ZFAS1 and MAPK pathways, western blot was used to detect the phosphorylation levels of ERK and p38 in the cells that were stably transfected with si-ZFAS1. As shown in the Figure 3D,E, the relative level of ERK phosphorylation in the ZFAS1 interference group was (1.412 ± 0.063), which was not significantly different from the control group (1.522 ± 0.052) (*P* > 0.05), and the p38 phosphorylation level decreased by 0.56 times (*P* < 0.01) after TNF-α induced apoptosis. The expression of ZFAS1 was decreased following inhibition of p38. Therefore, ZFAS1 was involved in the anti-apoptotic process induced by pORF5 through the MAPK/p38 pathway.

## Discussion

Genital tract infection of *C. trachomatis* had become one of the leading factors of sexually transmitted infections (Karim et al., 2018). However, the interaction between *C. trachomatis* and host remains to be clearly determined. In the past decade, lncRNAs and mRNAs expression profiles have been used widely to uncover the potential molecular mechanisms contributing to the pathogenesis of many diseases, such as cancers, viral infections, and some intracellular bacterial infections. Many studies have shown that pORF5 protein is the main virulence factor of *C. trachomatis* and plays an important role in the pathogenesis of *C. trachomatis* (Chen et al., 2010). In this study, we investigated the expression of lncRNAs and mRNAs in pORF5-transfected HeLa cells. Compared with the GFP-transfected cells, the expression profiles of lncRNAs and mRNAs were obviously altered in pORF5-transfected cells. GO and KEGG pathway analyses of the differentially expressed mRNAs implied some of the underlying functions and pathways associated with the pathogenesis of *C. trachomatis* infection. Here, we confirmed that pORF5 protein up-regulates lncRNA ZFAS1 to activate the MAPK/p38 pathway and promote host cell survival.

ZFAS1 is an antisense lncRNA to the 5′ end of the protein-coding gene *ZNFX1*, which is identified to be involved in many signaling transduction pathways, such as cell cycle, Wnt/β-catenin pathway and PI3K/Akt pathway, etc. (Fan et al., 2018; Xu et al., 2018; Liu et al., 2019). Its role in cells is related to promoting the cell cycle and inhibiting apoptosis, and is considered to be an oncogene. ZFAS1 is also predicated to be a prognostic biomarker for many diseases (Feng et al., 2019). We screened ZFAS1 by lncRNA microarray, and carried out follow-up studies. ZFAS1 was up-regulated in pORF5-transfected cells. The knockdown of this lncRNA increased the apoptotic rate of pORF5-transfected cells. These data indicate that pORF5 secreted by *C. trachomatis* can regulate ZFAS1. ZFAS1 plays a role in the anti-apoptotic process of *C. trachomatis*.

Members of the MAPK family can be activated in response to a variety of stimuli, including three pathways, ERK, p38, and JNK, which play important roles in cell activation, stress response, cell differentiation and growth (Lee et al., 2020). The most significantly differentially expressed molecule MAPKAPK3 (Table 3) has been shown to play an essential role in MAPK pathway, which is consistent with the result that pORF5 activated the MAPK/ERK and p38 pathways. Meanwhile, our previous work showed that pORF5 could activate the MAPK/ERK pathway to inhibit apoptosis by up-regulating DJ-1 (Luo et al., 2020), and induce the production of pro-inflammatory cytokines by activating the MAPK/ERK and p38 signaling pathway (Zhou et al., 2013). MAPKAPK3 has been shown to interact with MAPK14, which also called p38α, a prototypic member of the p38 MAPK family. Many studies have also demonstrated that p38 plays a role in anti-apoptotic process (Qi et al., 2014; Chang et al., 2017). Many transcription factors encompassing a broad range of actions have been shown to be phosphorylated and subsequently activated by p38. Examples include activating transcription factor 1, 2, and 6 (ATF-1/2/6), p53, MITF1, DDIT3, ELK1, and high mobility group-box protein 1 (HMGB1) and so on (Zarubin and Han, 2005). Consistent with the changes of these transcription factors, pORF5 protein was proved to be able to up-regulate DDIT3, activate the expression of unfolded protein and its downstream molecules ATF4, ATF6, and CHOP, thereby promoting cell autophagy (Wen et al., 2020), and also up-regulate the expression of HMGB1 and induce mitochondrial autophagy to resist apoptosis (Lei et al., 2017). MAPK pathway deeply influences the cellular physiological processes, and can active by pORF5 secreted by *C. trachomatis*.

Recent research has reported that influenza A strain H1N1 infection upregulated ZFAS1 expression. This lncRNA harbored small open reading frames, which potentially encode for micropeptides, and was predicated to play roles in stress induction (Razooky et al., 2017). Consistent with this, the result of GO analysis of upregulated mRNAs showed that the most three significant term of biological processes were response to endoplasmic reticulum stress, unfolded protein, and endoplasmic reticulum unfolded protein response, which were typical processes of stress for intracellular environment recovery. Our previous research has reported that pORF5 can activate unfolded protein response to induce autophagy via MAPK/ERK signaling pathway to protect cells. Similar to this result, pORF5 was proved to activate MAPK/ERK and MAPK/p38, and increase ZFAS1 to against apoptosis via MAPK/p38. The potential role of ZFAS1 micropeptides in chlamydial infection, especially in ER-stress induced by *C. trachomatis* requires further study.

Studies have reported that *Chlamydia pneumonia* can induce ET-1 production through the MAPK/p38 signaling pathway in vascular smooth muscle cells to promote cell proliferation (Kern et al., 2009). ZFAS1 has been shown to activate MAPK signaling pathway, and promote the migration and invasion of cervical cancer cells by regulating the MAPK/p38 signaling pathway (Gan et al., 2019; Han and Shen, 2020). In our experiment, MAPK/p38 activation was repressed during the anti-apoptotic process after interfering ZFAS1 expression, indicating that ZFAS1 can exert an anti-apoptotic effect by regulating the p38 signaling pathway. Meanwhile, the usage of p38 pathway inhibitors reduced the anti-apoptotic effect of pORF5-transfected cells, and decreased the progeny of *C. trachomatis*. A study in herpes simplex virus type 1 (HSV-1) showed that HSV-1 induced MAPK/p38 activation during infection, and utilized this pathway to enhance transcriptions of specific viral gene promoters, thereby increasing viral yield (Zachos et al., 2001). Similar to the virus, *C. trachomatis* is a strictly intracellular pathogen. Therefore, p38 may also play an important role in the intracellular replication of *C. trachomatis*. The next step is to study how lncRNA gathers through the p38 signaling pathway to regulate UPR and HMGB1 to participate in autophagy and anti-apoptotic processes, and which will further improve the understanding of the intracellular survival mechanism of *Chlamydia*.

In summary, we demonstrated that pORF5 altered the expression of lncRNAs and mRNAs in pORF5-transfected HeLa cells, and can activate the p38 pathway by up-regulating ZFAS1, promoting the survival of host cells and the proliferation of *C. trachomatis*. Our study may help to understand the interplay between lncRNAs and coding genes anticipated in the pathogenesis of *C. trachomatis*.

## Data Availability Statement

The datasets presented in this study can be found in online repositories. The names of the repository/repositories and accession number(s) can be found below: https://www.ncbi.nlm.nih.gov/, GSE154341.

## Author Contributions

SS: data curation. HC: formal analysis and writing – review and editing. ZL: funding acquisition. YZ: investigation. LZ: methodology. QH and ZL: resources and supervision. YW: software and writing – original draft. MS and SS: validation. YW, FL, and MS: visualization. All authors contributed to the article and approved the submitted version.

## Conflict of Interest

The authors declare that the research was conducted in the absence of any commercial or financial relationships that could be construed as a potential conflict of interest.

## Footnotes

1 http://davia.abcc.ncifcrf.gov/

2 http://www.genome.jp/kegg

3 https://string-db.org/

4 https://cytoscape.org/

5 https://metascape.org/

## Supplementary Material

Supplementary Figure 1 Overview of differentially expressed lncRNAs and mRNAs. **(A,B)** The heat map of detected lncRNAs **(A)** and mRNAs **(B)**. **(C,D)** The scatter plot of detected lncRNAs **(C)** and mRNAs **(D)**.

Supplementary Figure 2 Classification of differential lncRNAs. **(A)** The distribution of differentially expressed lncRNAs in chromosomes. **(B,C)** The classification of differentially expressed lncRNAs.

Supplementary Figure 3 Validation of the lncRNA and mRNA expressions. **(A,B)** The validation of differentially expressed mRNAs **(A)** and lncRNAs **(B)** by qRT-PCR.

Supplementary Figure 4 GO and KEGG analysis of differentially expressed mRNAs. **(A,C,E)** The analysis of GO biological process **(A)**, cellular components **(C)**, and molecular function **(E)** of up-regulated mRNAs. **(B,D,F)** The analysis of GO biological process **(B)**, cellular components **(D)**, and molecular function **(F)** of down-regulated mRNAs. **(G)** The analysis of KEGG pathways of up-regulated mRNAs. **(H)** The analysis of KEGG pathways of down-regulated mRNAs.

Supplementary Figure 5 The Protein-protein interaction net (PPI) network of differentially expressed mRNAs.
