# Supplementary figures and images for: Chlamydia trachomatis Plasmid Protein pORF5 Up-Regulates ZFAS1 to Promote Host Cell Survival via MAPK/p38 Pathway
Source: Front Microbiol. 2020 Dec 17;11:593295. doi: 10.3389/fmicb.2020.593295 (PMC7773608; doi:10.3389/fmicb.2020.593295)

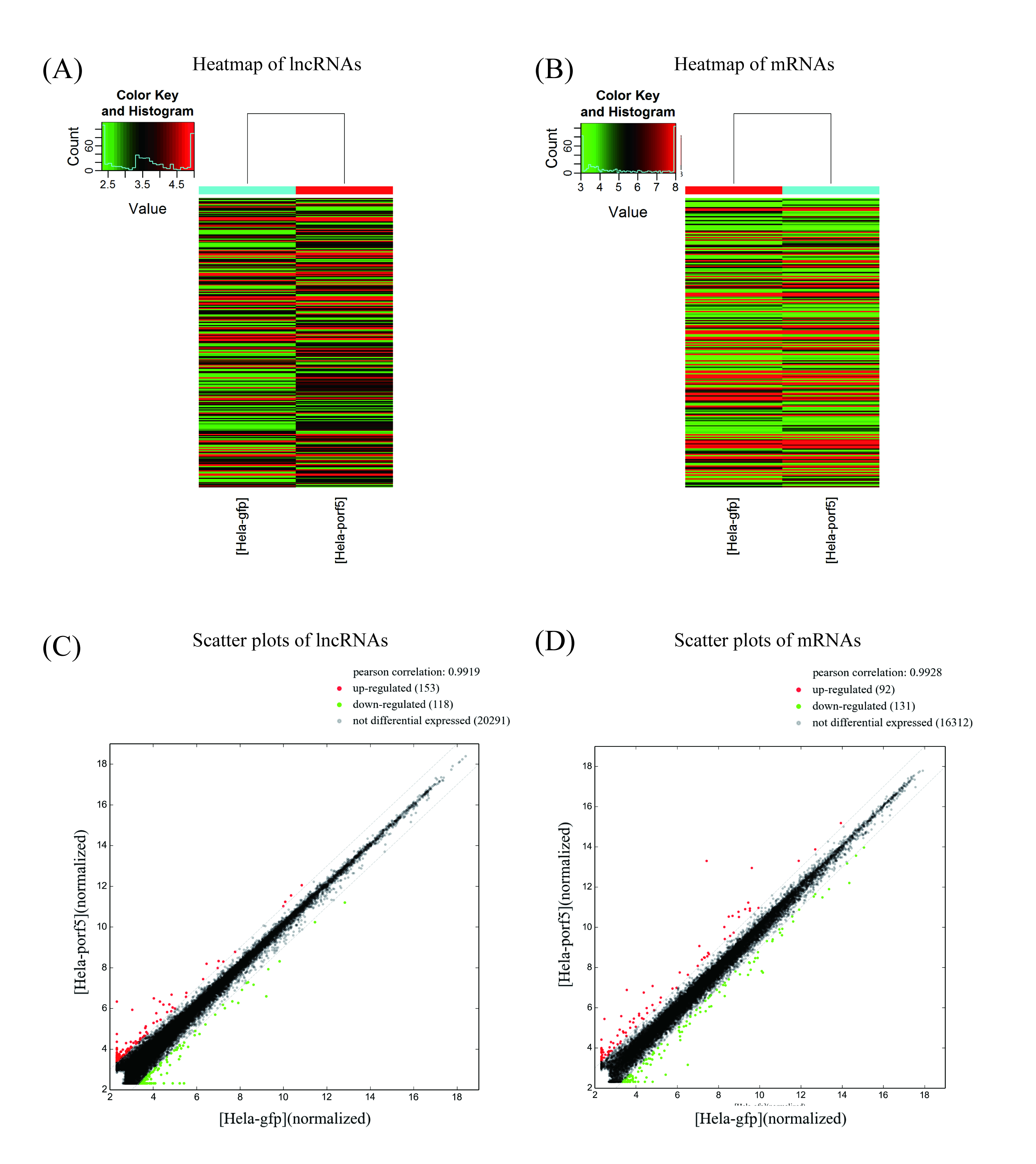

Supplement: Supplementary Figure 1 — Overview of differentially expressed lncRNAs and mRNAs. (A,B) The heat map of detected lncRNAs (A) and mRNAs (B). (C,D) The scatter plot of detected lncRNAs (C) and mRNAs (D). [file Image_1.tif]

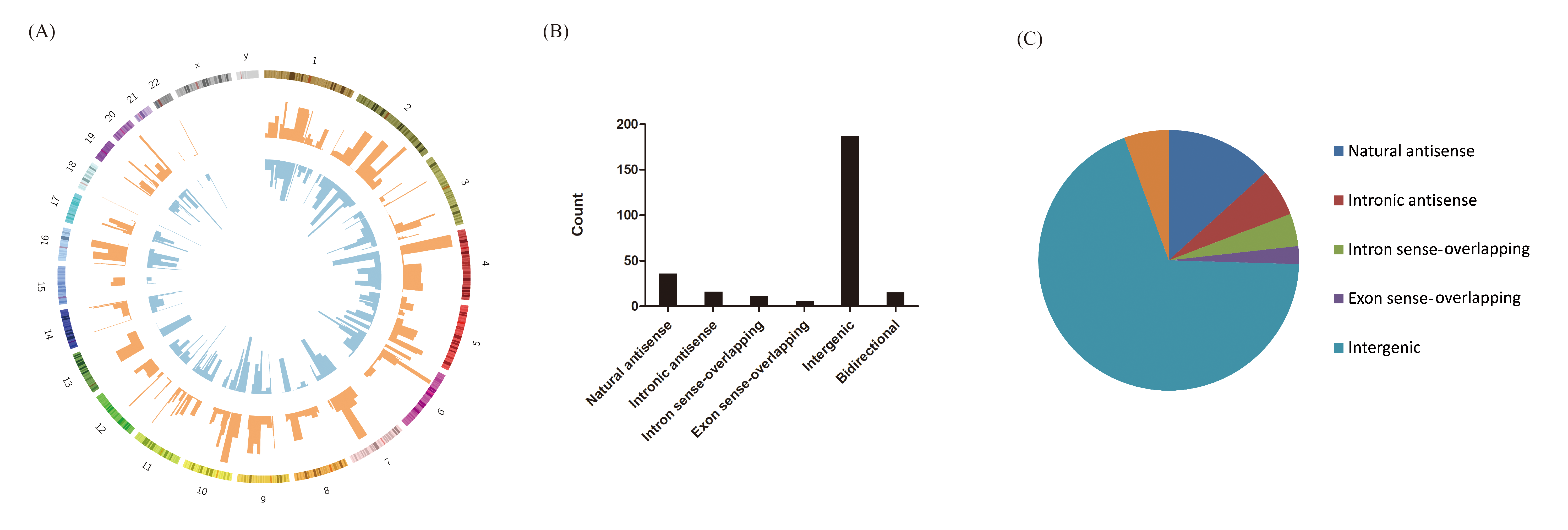

Supplement: Supplementary Figure 2 — Classification of differential lncRNAs. (A) The distribution of differentially expressed lncRNAs in chromosomes. (B,C) The classification of differentially expressed lncRNAs. [file Image_2.tif]

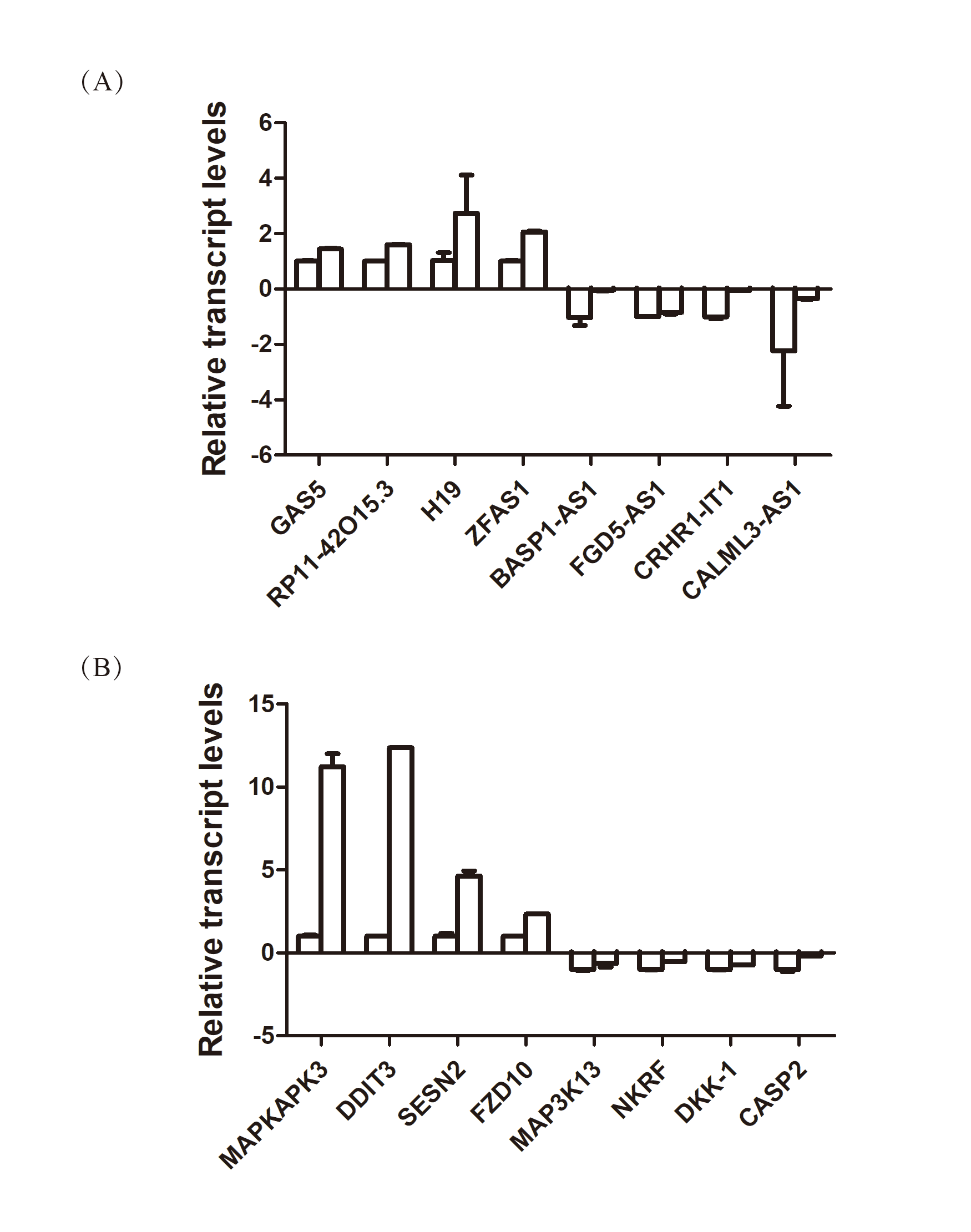

Supplement: Supplementary Figure 3 — Validation of the lncRNA and mRNA expressions. (A,B) The validation of differentially expressed mRNAs (A) and lncRNAs (B) by qRT-PCR. [file Image_3.tif]

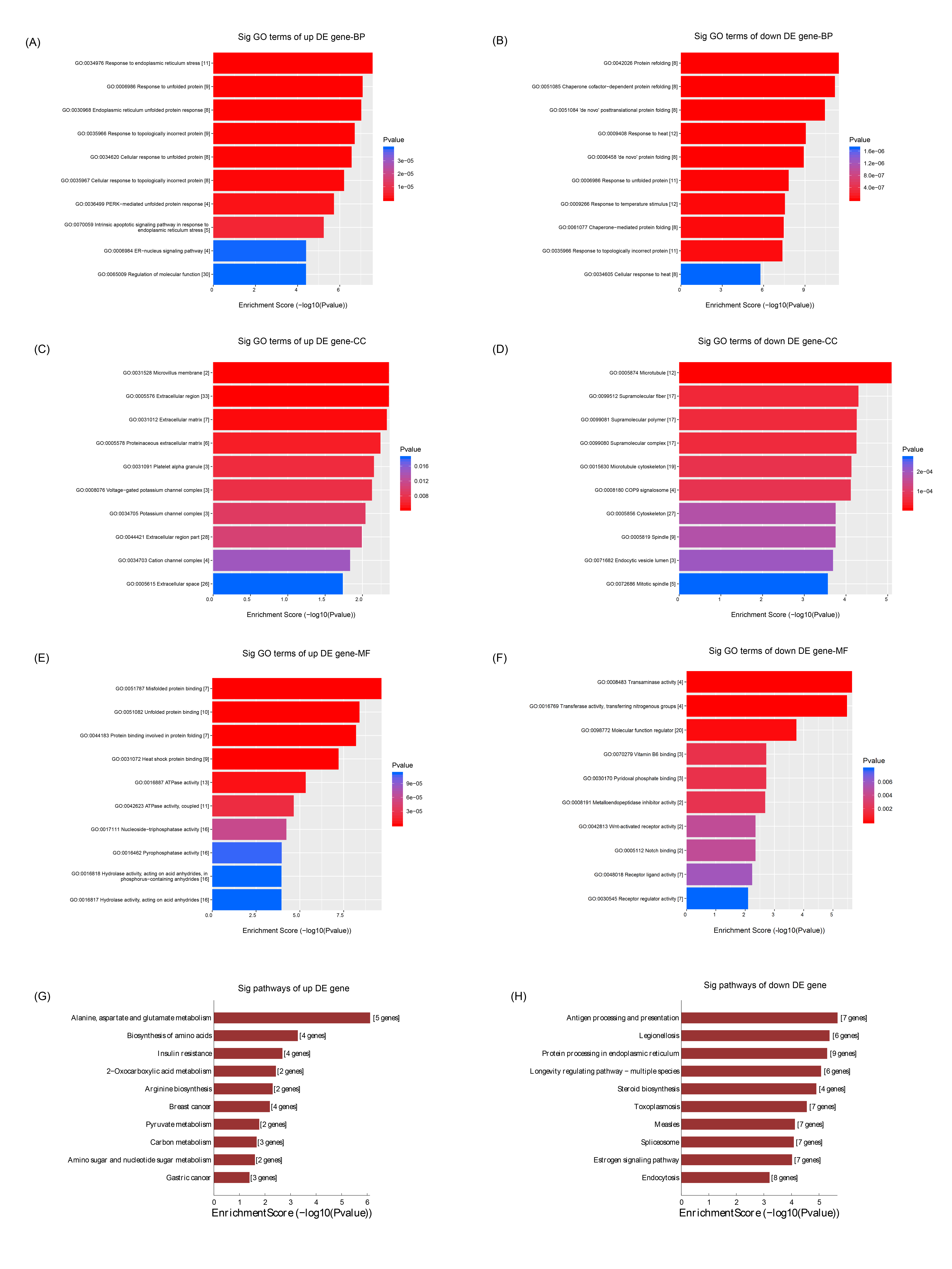

Supplement: Supplementary Figure 4 — GO and KEGG analysis of differentially expressed mRNAs. (A,C,E) The analysis of GO biological process (A), cellular components (C), and molecular function (E) of up-regulated mRNAs. (B,D,F) The analysis of GO biological process (B), cellular components (D), and molecular function (F) of down-regulated mRNAs. (G) The analysis of KEGG pathways of up-regulated mRNAs. (H) The analysis of KEGG pathways of down-regulated mRNAs. [file Image_4.tif]

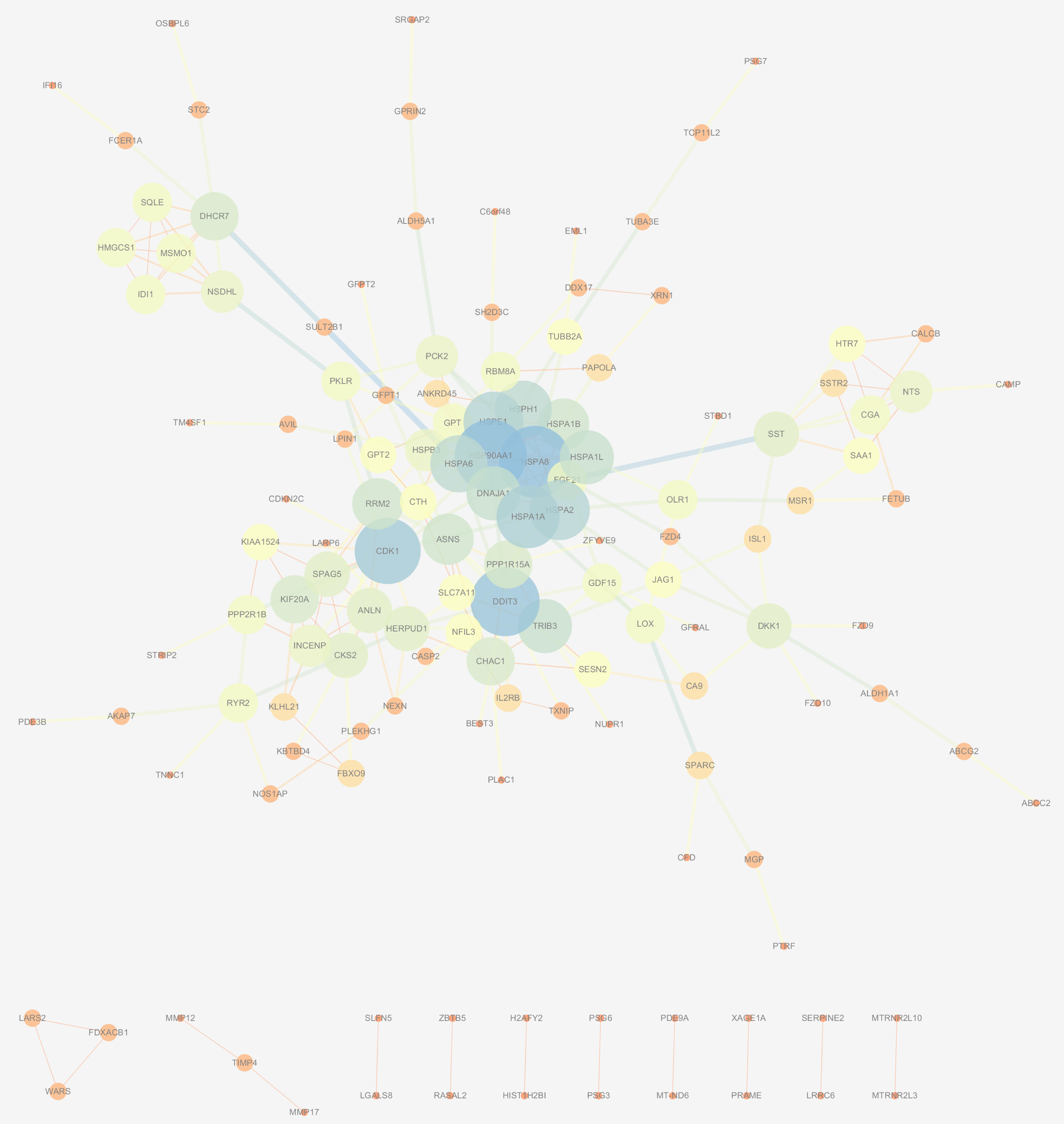

Supplement: Supplementary Figure 5 — The Protein-protein interaction net (PPI) network of differentially expressed mRNAs. [file Image_5.tif]
